# The impact of digital financial inclusion on bank performance: An exploration of mechanisms of action and heterogeneity

**DOI:** 10.1371/journal.pone.0309099

**Published:** 2024-08-20

**Authors:** Jing Zhao, Congqi Wang, Haslindar Ibrahim, Yanpeng Chen

**Affiliations:** School of Management, Universiti Sains Malaysia, George Town, Malaysia; BeiHang University School of Economics and Management, CHINA

## Abstract

The use of digital technology by banks and other financial institutions to facilitate financial inclusion is referred to as digital financial inclusion. This fusion of digital finance and traditional banking methods has the potential to impact banks’ operational effectiveness. This study uses the panel effects model to examine the link between digital financial inclusion and bank performance in 30 Chinese provinces from 2012 to 2021. This research uses kernel density estimation to examine the spatial-temporal growth patterns of both variables. The mediator variable in examining how digital financial inclusion affects bank performance is risk-taking. Finally, the paper analyses the regional heterogeneity of the impact. It presents the following conclusions: (1) In China, digital financial inclusion and bank performance have constantly increased, with noticeable regional variances in their development levels. This regional inequality has widened gradually since 2018, yet it has not resulted in polarization. (2) The significant positive correlation between digital inclusive finance and banking performance indicates that banking performance tends to increase with the enhancement of digital inclusive finance. (3) Digital financial inclusion impacts bank performance, with risk-taking as a moderator. The spread of digital financial inclusion services enhances banks’ willingness to take risks, enhancing overall efficiency. (4) Digital financial inclusion boosts bank performance in the Northwest, South, North, and East regions while lightly inhibiting it in the Central region. Based on the findings, this study makes bank and government suggestions.

## 1. Introduction

Digital financial inclusion is a type of inclusive finance that has emerged due to ongoing advancements and innovations in digital technology and science. Its primary objective is to offer high-quality financial products and services to individuals from all social classes and industries. This entails expanding upon the previous modes of financial operations by overcoming the limitations of low efficiency in credit and business processing caused by geographical barriers and information disparities. By doing so, digital financial inclusion aims to facilitate the overall economic development of different regions and industries while enhancing individuals’ living standards [1, 2]. Han, Zeng [3] argue that the financial inclusion of resources and cost consumption in daily operations is a significant concern that warrants careful consideration. This is due to the considerable societal demand for digital financial inclusion and the increasing range of areas in which financial products and services are expected to meet such demand.

The use of digital technology infrastructure in banks and other financial institutions has deepened in recent years, and digital technology, primarily big data, cloud computing, and AI, can be seen everywhere in people’s daily lives and has gradually penetrated the financial sector. The degree of application of such technologies in the financial sector determines the degree of convenience in updating financial products and services, affecting the customer’s experience of the products and satisfaction with the services [4]. Li, Long [5] underscore the current landscape of digital financial inclusion as a culmination of the historical trajectory of microfinance enterprises, ultimately transforming into a tangible expression of inclusive finance. Additionally, given their status as the earliest financial institutions in China, banks hold an indispensable and integral position within the present financial ecosystem. According to Wang, Zhang [6], the performance of banks is influenced by a myriad of factors owing to the diverse financial resources within society and the perpetual challenge of maintaining stable levels of consumption and demand over time. However, the effective deployment of digital infrastructure and the successful implementation of financial inclusion strategies have consistently positively impacted bank performance. This improvement in bank performance underscores the efficacy of financial inclusion policies and reflects the extensive endeavours aimed at its promotion.

Consequently, implementing conventional inclusive financial services by commercial banks has increased workforce, risk, and operations costs. Suppose commercial banks continue to allocate more of their financial resources to clients with long-tail needs. This conclusion would unavoidably impact the allocated resources for top-tier clients, ultimately resulting in an inability to meet the financial service requirements of any specific demographic effectively. Consequently, the decline in the performance of commercial banks is growing more severe. The establishment of digital inclusive finance directly resulted from integrating financial science and technology into the inclusive finance sector. This integration was made possible by the creation and growth of the Internet financial market. Inclusive financial services have broadened their availability and advantages for a substantial number of financial consumers. The integration of digital technology into conventional inclusive financial services has significantly enhanced the efficiency of financing. Nevertheless, the emergence of digital financial inclusion has brought about both obstacles and hazards while presenting commercial banks with prospects for growth. Although the digital financial inclusion model has successfully stimulated the enhancement of bank performance and decreased the expenses related to financial services for commercial banks, it is crucial to acknowledge the emerging risks that banks are currently vulnerable to and the potential market rivalry.

Therefore, investigating the influence of digital finance on bank performance not only enhances the research in relevant domains but also holds significant practical implications for enhancing bank performance. Most prior research on digital financial inclusion primarily examines the impact on individuals’ lives and entrepreneurship. However, there needs to be more research investigating the relationship between digital financial inclusion and bank performance. Furthermore, the existing studies tend to adopt a macro perspective, and there is a scarcity of literature that quantitatively explores the direct influence of digital financial inclusion on bank performance and analyses any variations in this relationship. This paper uses empirical analysis to examine the impact of digital financial inclusion on bank performance. The study employs the kernel density estimation model to investigate the spatial-temporal development trend and characteristics of digital financial inclusion and bank performance.

Additionally, the ordinary panel effect model is used to analyse the impact of digital financial inclusion on bank performance in detail. Furthermore, the mediation effect model is utilized to analyse the impact of digital financial inclusion on bank performance, with risk-taking serving as a mediator variable. The mediation effect model is again employed to analyse how digital financial inclusion affects bank performance. Additionally, the study investigates the regional heterogeneity of the influence of digital financial inclusion on impression performance across different areas. The research presented in this paper enhances the existing research in relevant disciplines and establishes a theoretical foundation for understanding the process via which digital inclusive finance affects bank performance.

## 2. Literature review

Xiao [7] contends that implementing inclusive financial policies in China has successfully reduced income inequality and addressed the disparity between urban and rural growth. Chinese banks have a crucial role in the country’s financial sector, aggressively promoting the development of inclusive financial services. By incorporating digital technology into their operations, banks can overcome the constraints of the traditional financial model, optimizing the effectiveness of financial inclusion initiatives [8]. Based on the previous studies, digital financial inclusion may impact bank performance regarding the breadth of coverage, depth of use, and degree of digitization [9, 10]. The breadth of coverage primarily pertains to the bank’s utilization of big data and other digital technologies to enhance the coverage of areas and industries not typically addressed by conventional financial services. It also aims to strengthen the coverage of areas already addressed by traditional financial services. Depth of use pertains to how much the audience embraces inclusive financial policies following their widespread adoption. This includes residents’ use of digital financial products and services, the frequency of their usage, and other activities that can impact the bank’s performance [11, 12]. On the one hand, banks employ big data and artificial intelligence technology to reach out to rural and remote areas not served by traditional financial services because of time and space constraints. This greatly expands the audience and potential clientele for the banking industry and, on the other hand, broadens the opportunities for profit from the bank’s performance and raises the probability of the bank’s success [13, 14]. On the other hand, Wang, Yuan [15] argued that small and medium-sized businesses’ financing problems could be effectively resolved by implementing digital financial inclusion policies and implementing banks and other financial institutions effectively. Expanding digitalization can boost rural area access to financial services by expanding bank branch ability to provide inclusive banking and making business processes and audits more easily accessible [16, 17]. From the preceding research, bank performance may be boosted considerably by increasing the extent to which digital inclusion is used, the breadth of coverage, and the level of digital application in financial services. This allows us to deduce the study’s first hypothesis:

**H1: Digital financial inclusion positively affects bank performance.**

According to Jing, Miao [18], bank performance may show phases of localized stagnation or regression since they are subject to various risks in their operations and services, some of which can be predicted and prevented, while others are unpredictable. Improving the bank’s risk-taking capability entails increasing the sorts of risks that can be averted and dealt with and decreasing the types of risks that cannot be forecast and prevented in order to ensure the bank’s performance grows gradually [19, 20]. With the increasing adoption of digital technology in the realm of financial inclusion, financial institutions such as banks have the opportunity to optimize their online service procedures, enhance their capacity to assume risks in their day-to-day activities and reduce avoidable losses resulting from ineffective employee conduct [21, 22]. Additionally, it is anticipated that financial institutions across various regions will continue to elevate the standard of digital technology innovation and financial inclusion. This will generate intense market competition, leading to the complete implementation of advanced technological methods by various financial institutions and products [23–25]. According to Bhaskaran, Chang [26], expanding digital financial inclusion has numerous benefits. It enables individuals in underserved areas, such as rural areas, to access financial products tailored to their needs. Additionally, it assists small and medium-sized businesses overcome growth barriers caused by limited capital. Furthermore, it allows banks to diversify their sources of revenue. Considering China’s significant rural population, digital financial inclusion can effectively address their needs while mitigating the risk banks encounter when engaging in credit business. Thanks to big data and other digital technologies, financial institutions can now accurately forecast customer credit and spending limits [27]. An improved ability to take on risk when faced with the threat of bad debts in credit operations is another benefit of a bank’s diversified revenue streams [28, 29]. The above investigation establishes the second hypothesis of this study:

**H2: There is a mediating effect of digital financial inclusion on bank performance with risk-taking as the mediating variable.**

Since the use of digital technology in banking has increased the bank customer base and thus improved the bank performance, and since the degree to which digital technology has been developed and is being used has a significant impact on the bank performance, banks and other financial institutions have begun to invest heavily in the Internet and the innovation of digital technology [30–32]. However, Xu [33] argues that the current state of China’s digital technology growth demonstrates that the regional development imbalance persists and that this causes apparent inequalities in the economic activities of the regions. It is clear that differences in the development of digital technology in different regions are at the root of variations in the level of bank digitization and that these variations, in turn, affect the breadth of coverage and depth of use of financial inclusion, as is most obviously seen in the high level of digital technology in the region, the bank in the implementation of financial inclusion policy will cover a more comprehensive array of people [34, 35]. It has been found that the positive effect of digital financial inclusion on bank performance is more significant in China’s southeast coastal region than in the northwest and that this may be due to the southeast coastal region’s higher level of economic development, a strong capacity for innovation in digital technology, and a greater degree of integration with the financial industry [36]. The growth of digital finance, Diniz, Birochi [37] argue, may also hurt bank performance. Local banks are smaller in scale and slower in capital operation, so their ability to prevent and control this impact could be more robust, and their performance may show more apparent fluctuations. This is because larger banks, such as state-owned banks, have more channels for capital accumulation and can better withstand this negative impact and minimize the fluctuation of bank performance. There is also regional variation in terms of bank size. The above analysis leads to the third hypothesis of this study:

**H3: There is regional heterogeneity in the impact of digital financial inclusion on bank performance.**

## 3. Research design

### 3.1 Research methodology

#### 3.1.1 Kernel density estimation

Kernel density estimation is mainly a nonparametric estimation method used to estimate the distribution density function, which is more intuitive than the histogram and is a further abstraction of the histogram [38]. Therefore, this paper uses the kernel density estimation method to plot the kernel density estimation of bank performance and digital financial inclusion, respectively, which is calculated as follows:

$${\hat{f}}_{h}(x)=\frac{1}{nh}\sum_{i=1}^{n}K(\frac{x-x_{i}}{h})$$ (1)

Where x denotes the point to be estimated, *x*_*i*_ denotes the sample point selected in this study, and *K*((*x*−*x*_*i*_)/*h*) refers to the kernel function that contains the parameter *h*. In general, when the parameter *h* of this kernel function is greater than 0, it is referred to as the bandwidth, and the value of the bandwidth *h* affects the degree of smoothing as well as the estimation accuracy of this kernel function.

#### 3.1.2 Ordinary panel effect model

Given that understanding the impact of digital financial inclusion on bank performance is essential for this study, the paper begins by regressing the direct impact of digital financial inclusion on bank performance using the ordinary panel effect model. The formula for its calculation is as follows:

$$BP_{it}=\alpha_{0}+\alpha_{1}DFI_{it}+\sum_{j=2}^{6}\alpha_{j}control_{it}+\gamma_{i}+\mu_{t}+\varepsilon_{it}$$ (2)

Where *DFI*_*it*_ refers to the level of digital financial inclusion in city *i* in year *t*, *BP*_*it*_ refers to the level of bank performance in city *i* in year *t*, *control*_*it*_ refers to the level of development of the control variables in this paper in city *i* in year *t*, *γ*_*i*_ refers to time fixed effects, *μ*_*t*_ Refers to the individual fixed effects, *ε*_*it*_ denotes the random disturbance term of the model, and *α*_0_ denotes the coefficient of return of the regression test model.

#### 3.1.3 Mediation effects model

Based on the analysis of Hypothesis 2, banks and other financial institutions can enhance their risk-taking capacity through technological innovation and expanding revenue channels. This can help mitigate default and bad debt in their credit business and ensure operational efficiency. Thus, this paper refers to the research approach of Hong, Xuefei [39] to take risk-taking as a mediating variable and build a mediation effect model in order to verify Hypothesis 2 and explore whether there is a mediating variable of risk-taking in the mechanism of digital financial inclusion on bank performance. Here is the exact formula for calculating it:

$$BP_{it}=\alpha_{0}+\delta DFI_{it}+\sum_{j=1}^{6}\beta_{j}control_{it}+\gamma_{i}+\mu_{t}+\varepsilon_{it}$$ (3)

$$TR_{it}=\alpha_{0}+\lambda DFI_{it}+\sum_{j=1}^{6}\beta_{j}control_{it}+\gamma_{i}+\mu_{t}+\varepsilon_{it}$$ (4)

$$BP_{it}=\alpha_{0}+\theta DFI_{it}+\eta TR_{it}+\sum_{j=1}^{6}\beta_{j}control_{it}+\gamma_{i}+\mu_{t}+\varepsilon_{it}$$ (5)

In the above equation, *TR*_*it*_ denotes the level of risk taking in province *i* in year *t*. The regression coefficient *δ* in Eq (3) is the total effect of the impact of digital financial inclusion on bank performance. The regression coefficient *λ* in Eq (4) is the effect of digital financial inclusion on risk-taking. The regression coefficient *θ* of Eq (5) is the direct effect of the impact of digital financial inclusion on bank performance, whereas the coefficient *η*×*λ* of the regression coefficient *η* in Eq (5) multiplied by the regression coefficient *λ* in Eq (4) is the indirect impact effect of the impact of digital financial inclusion on bank performance, the abbreviations of the other indicators in the formula are interpreted in the same way as in Eq (2).

### 3.2 Variable selection and data sources

#### 3.2.1 Selection of variables

*1*. *Dependent variable*. Banks are the most frequently used financial institutions in people’s daily lives; therefore, the performance level of banks can reflect the living standards and economic conditions of residents, and at the same time, the performance level of banks can answer the degree of implementation of digital financial inclusion policies. Increased digitization and financial product innovation can always have a beneficial effect on bank performance, and the degree to which a bank invests in digital financial inclusion can be seen in the degree to which its relevant financial goods and service operations are digitized. Therefore, this research aims to examine the determinants of bank performance. Based on Rickinghall [40] analysis of the available literature, the return on equity (ROE) metric was selected to represent the level of bank performance.


$$ROE_{it}=Npro_{it}÷Sret_{it}$$ (6)

$$ROA_{it}=Npro_{it}÷Tass_{it}$$ (7)

In the above equation, *ROE*_*it*_ and *ROA*_*it*_ denote the return on net assets and return on total assets in year *t* of province *i*, respectively. Where *Npro*_*it*_ denotes the net profit of province *i* in year *t*, net profit is the profit obtained by subtracting total costs and expenses from the total income of all commercial banks in the region during the year. *Sret*_*it*_ denotes the net assets of province *i* in year *t*, and its amount is the balance of assets minus liabilities. *Tass*_*it*_ denotes the total assets of province *i* in year *t* and is the sum of the values of all assets of the firm in the same period.

*2*. *Independent variable*. With the deep integration of digital technology and inclusive finance, the development of digital inclusive finance has positively impacted the performance levels of banks. Conversely, the improvement in bank performance may also influence the development of digital inclusive finance, enhancing its breadth of coverage and depth of usage. These two factors are interrelated and interact with each other. Therefore, this paper takes digital inclusive finance as the core explanatory variable to explore its specific impact on bank performance. According to relevant literature, Peking University has constructed 33 related indicators based on the breadth of coverage, depth of usage, and digitalization level of digital inclusive finance, which accurately reflect China’s level of development in digital inclusive finance, the specific construction is shown in Table 1. Hence, this method is used in this paper to measure China’s development level in digital inclusive finance [41, 42].

**Table 1:** Evaluation indicators system of digital inclusive finance.

Primary indicators	Secondary indicators	Tertiary indicators	Specific indicators
Digital Inclusive Finance Index	Breadth of digital financial inclusion coverage	Account coverage ratio	Number of Alipay accounts per ten thousand people
			The proportion of Alipay users who have linked their bank cards
			The average number of bank cards linked to each Alipay account
		Payment business	Number of payments per capita
			Amount paid per capita
			Number of high-frequency active users as a proportion of annual active 1 time or more
		Money fund operations	The average number of transactions per person buying Yu’ebao
			Average amount of money spent per person on Yu’ebao
			The number of Yu’ebao purchasers per ten thousand Alipay users
	Depth of use of digital financial inclusion	Personal consumption loan	In every ten thousand adult users of Alipay, the number of users using internet consumption loans
			Number of loans per capita
			Amount of loans per capita
		Small and micro-operator	Number of internet-based micro-enterprise loan users per ten thousand adult Alipay users
			Average number of loans per household for micro and small operators
			Average loan amount for micro and small operators
		Insurance business	Number of people involved in Internet investment and wealth management per 10,000 Alipay users
			Average number of investments per capita
			Amount of investment per capita
		Investment business	Number of people involved in Internet investment and wealth management per 10,000 Alipay users
			Average number of investments per capita
			Amount of investment per capita
		Credit business	Average number of credit inquiries per capita for individuals
			Number of users utilizing credit-based services per 10,000 Alipay users
	Degree of digitalization in digital inclusive finance	Mobility	Percentage of mobile payment transactions
			Percentage of mobile payment amount
		Cost-effective	Average lending rate for micro and small operators
			Average personal loan interest rate
		Criticization	Percentage of transactions paid with Huabei
			Alipay Huabei payment proportion
			Sesame credit deposit-free transactions as a percentage of transactions requiring a deposit
			Sesame Credit deposit-free amount as a percentage of all amounts requiring a deposit
		Facilitation	Proportion of times users use QR codes for payment
			Percentage of amount paid by users using QR codes

*3*. *Mediating variable*. The non-performing loan provision coverage ratio is an essential indicator for assessing a bank’s risk-bearing capacity. It directly impacts the bank’s performance. As the non-performing loan ratio increases, banks need to set aside more provisions to cover losses, leading to a decrease in profitability. Conversely, a decrease in the non-performing loan ratio results in a corresponding reduction in provisions, which positively impacts profitability. Moreover, the level of the non-performing loan provision coverage ratio effectively reflects the risk level of bank loans and the financial soundness of the bank. Therefore, this study adopts the method referenced by Wang and Wang [43], using the loan loss provision coverage ratio to measure banks’ risk-taking ability. This ratio primarily assesses the proportion of provisions set aside for managing loan default risks relative to the amount of non-performing loans. The provisions for managing loan default risks include general provisions, specific provisions, and special provisions. The classification of bank non-performing loans mainly includes substandard loans, doubtful loans, and loss loans.

*4*. *Control variables*. The performance level of banks in the actual operation process may be affected by many aspects, so this paper selects five indicators as the control variables of this study, namely economic development level, industrial agglomeration level, urbanisation level, bank net interest margin and capital adequacy ratio, respectively, from various aspects of society and bank development [44–46]. Precisely, the amount of economic progress is quantified by the logarithm of GDP per capita. The calculation of industrial agglomeration level involves the use of locational entropy. The calculation of the urbanization level involves determining the proportion of the urban population in the total regional population. The measurement of banks’ net interest margin involves calculating the difference between their interest income and interest expense as a percentage of interest-earning assets. The measurement of the capital adequacy ratio involves determining the proportion of capital to risk-weighted assets.

#### 3.2.2 Data sources

For this study, we first list all the relevant variables in China’s data from 2012 to 2021. Data pre-processing is conducted to eliminate provinces or data that do not meet our requirements but could still influence our conclusions. Ultimately, we agreed to select a sample size of 30 provinces in China. The primary data for each variable was collected from multiple publications, such as the China Statistical Yearbook, the China Industrial Statistical Yearbook, the China Population and Employment Statistical Yearbook, the China Financial Yearbook, provincial and municipal statistical yearbooks covering the period from 2012 to 2021, and the International Bureau of Statistics database accessible on their official website.

### 3.3 Variable data analysis

Table 2 presents the descriptive statistics for each variable, obtained from statistical analysis and econometric testing conducted using the Stata software in this research. The findings reveal notable disparities in bank performance among various locations in China. Specifically, the average bank performance is 13.311, with a standard deviation 5.876. Furthermore, the gap between the highest and lowest values is nearly 80-fold. The average of the digital financial inclusion index is 250.540, with a standard deviation of 87.744. The highest recorded value is 458.97, while the lowest is 61.47. The range of values is narrower than the disparity in performance of the region’s banks.

**Table 2:** Descriptive statistical analysis.

Variable	Obs	Mean	Std. dev.	Min	Max
**ROE**	300	13.311	5.876	0.401	31.659
**Digital Financial Inclusion Index**	300	250.540	87.744	61.47	458.97
**Economic development level**	9.325	0.463	8.598	10.780	9.325
**Degree of industrial agglomeration**	0.026	0.039	0.003	0.217	0.026
**Urbanization level**	0.602	0.118	0.363	0.896	0.602
**Bank net interest margin**	2.471	0.821	0.360	6.490	2.471
**Capital adequacy ratio**	13.390	2.342	7.000	33.350	13.390

Nevertheless, it continues to illustrate the imbalanced characteristics of the nation’s regional financial systems. The statistical results of each control variable, such as economic development level, degree of industrial agglomeration, urbanization level, bank net interest margin, and capital adequacy ratio, show variability across China’s provinces, indicating significant variations. In general, the statistical results suggest that the data for each variable falls within the normal range and does not contain any outliers. This makes the data appropriate for use as a foundation for empirical research.

**Fig 1:**
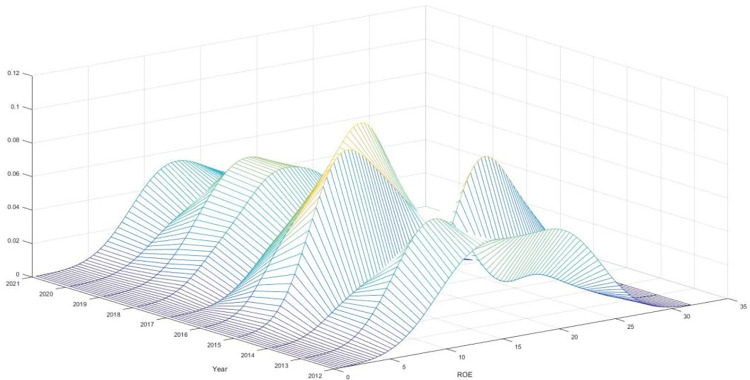
Estimated kernel density of bank performance.

**Fig 2:**
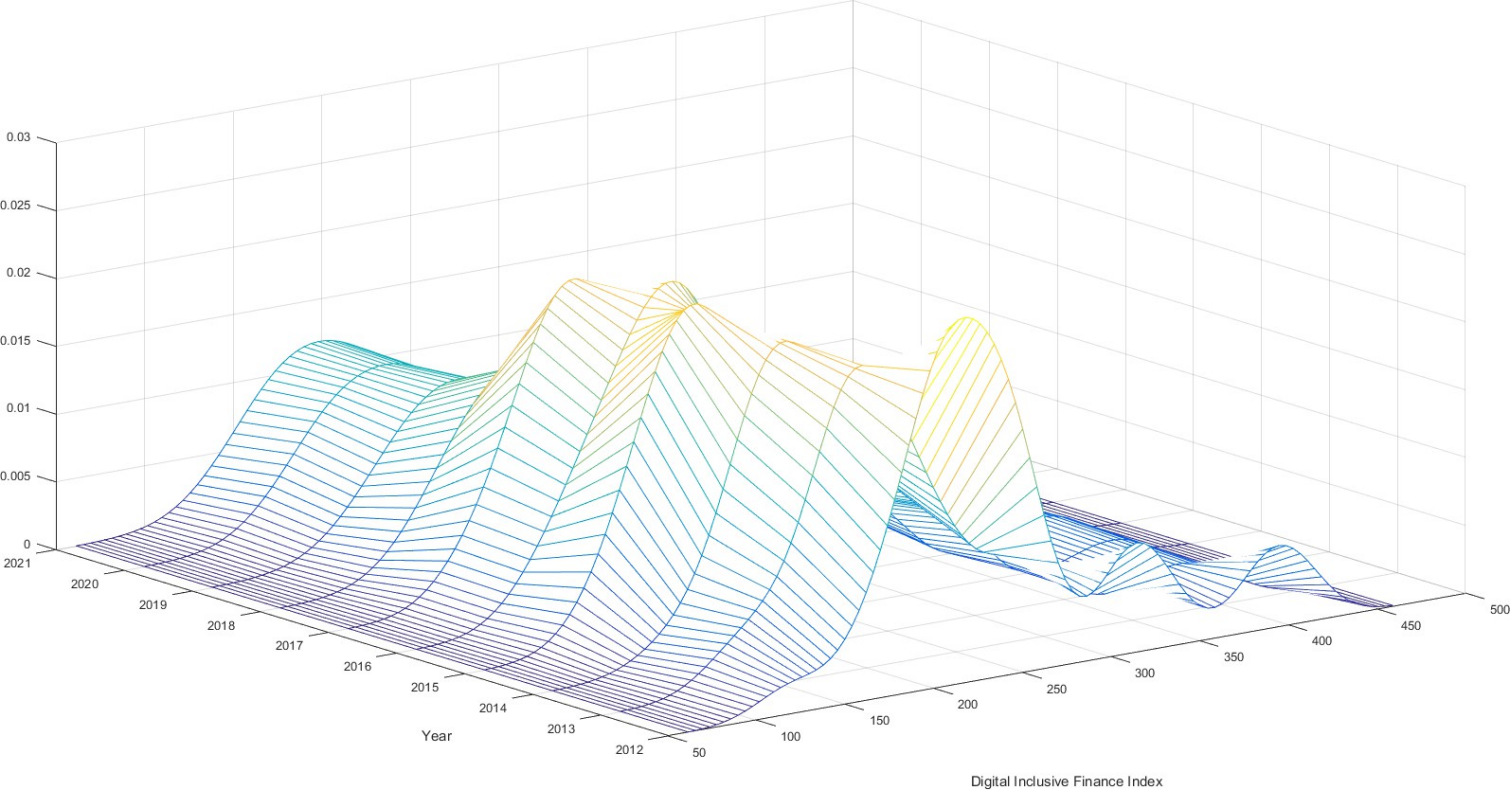
Estimated kernel density of digital financial inclusion.

The study utilizes the kernel density estimation approach to generate graphs representing the kernel density estimation of digital financial inclusion and bank performance. These graphs are displayed in Figs 1 and 2, respectively. The orientation of the central point of the kernel density curve, the existence of the trailing phenomena, and the alteration in the width of the peak wave can be utilized to infer the distribution and progression of digital financial inclusion and bank performance.

Fig 1 illustrates the changes in the kernel density curve of bank performance as time progresses. The observable shift towards the right in the kernel density curve suggests a clear improvement in bank performance. The bank performance varies greatly among China’s provinces, with certain provinces demonstrating significantly superior performance compared to other regions. This disparity can be attributed to the lagging phenomena shown in the kernel density curve of bank performance. This discovery provides additional evidence that a regional mismatch characterizes the evolution of China’s bank performance. Between 2012 and 2018, the peak of the kernel density curve that represents bank performance tended to change from a wide shape to a small one. These findings indicate that the extent of variation in bank performance between China’s provinces decreased dramatically throughout this period.

Nevertheless, the highest wave condition exhibited a pattern of transitioning from a more constricted state to a more expansive state between the years 2018 and 2021. Possible reasons for this phenomenon can be attributed to the initial phases of digital advancement, where banks worldwide needed to adopt digital technology adequately. The disparity in banking and credit services was primarily influenced by non-technical factors such as population concentration. Adopting digital technology in the financial sector will result in a widening revenue disparity between provinces with favourable geographical positions, advanced economic and scientific technologies, innovative solid capabilities, and slower economic development.

Fig 2 illustrates a kernel density curve representing the progress of digital financial inclusion. The curve shows a consistent rightward trend over time, indicating that China’s level of development in digital financial inclusion is also increasing. The presence of lagging phenomena is also apparent in the kernel density curve of digital financial inclusion, indicating differences in the amount of digital finance development among the provinces of China. Nevertheless, no discernible polarising pattern is evident in this fluctuation upon examining the quantity of wave crests. Furthermore, the degree of China’s digital financial inclusion development saw a minor increase from 2012 to 2017. However, starting from 2017, there has been a noticeable pattern of the gap widening each year until 2021. These results align with the findings of the wave crest analysis, which revealed that the wave crests of digital financial inclusion remained relatively stable from 2012 to 2017. A noticeable yearly pattern indicates a growing disparity in levels of development. In 2017, several affluent provinces experienced a surge in digital technological innovation. They increased the utilization of modern digital technology in the financial inclusion sector. However, the pace of digital technological innovation in poor regions could have been accelerated. This phenomenon can be attributed to the amalgamation of digital technology and financial inclusion, resulting in the emergence of digital financial inclusion enterprises.

## 4. Empirical analysis

### 4.1 Baseline regression analysis

This study utilizes Eqs (2)–(3) to create a regression model that examines the impact of the digital financial inclusion index on bank performance. The regression results are presented in Table 3. Model (1) displays the outcomes of regressing the digital financial inclusion index solely on bank performance, while model (2) showcases the outcomes of regressing the digital financial inclusion index on each of the control variables simultaneously. Upon examining the table, it is evident that conducting separate regressions for digital financial inclusion and bank performance yields a regression coefficient of 10.797 for digital financial inclusion. This coefficient is statistically significant at the 1% level, indicating that an increase in the level of digital finance development can significantly enhance bank performance. Specifically, for every unit of improvement in digital financial inclusion, the bank’s performance will increase by 10.797%. When digital inclusive finance is included in a regression analysis along with control variables and bank performance, the results show that digital inclusive finance has a significant positive impact on bank performance. This effect remains significant even when controlling for other factors, with a significance level of 1%. The regression results above confirm Hypothesis 1, which states that digital financial inclusion has a positive effect on bank performance. The advancement of digital financial inclusion will broaden the range of services offered by banks and increase the need for financial management and financing in remote areas and small and medium-sized enterprises. Additionally, the increased level of digitization will effectively lower the operating costs and credit costs of the bank, ultimately enhancing the bank’s performance.

**Table 3:** Analysis of baseline regression results.

Variables	Model (1)	Model (2)
	ROE	ROE
**Digital Financial Inclusion Index**	10.797***	5.848***
	(24.17)	(5.38)
**Economic development level**		-6.120***
		(-2.79)
**Degree of industrial agglomeration**		-2.210
		(-0.04)
**Urbanization level**		36.264***
		(3.19)
**Bank net interest margin**		-1.361***
		(-4.51)
**Capital adequacy ratio**		0.344***
		(3.62)
**Constant**	-72.127***	-11.116*
	(-29.55)	(-1.85)
**Observations**	300	300
**R-squared**	0.685	0.752
**Number of id**	30	30

Note: (1) T-statistics are in parentheses. (2) ***, **, and * indicate significant at the 1%, 5%, and 10% significance levels, respectively.

From the regression results of the control variables, the regression coefficients of the economic development level, the degree of industrial structure agglomeration and the bank net interest margin are all negative, but only the economic development level and the bank net interest margin pass the significance test at the 1% significance level, which indicates that the economic development level and the bank net interest margin show a significant negative impact on bank performance, mainly because with the economic development level and the degree of industrial agglomeration, it will The main reason is that as the level of economic development and industrial agglomeration increases, the financial market becomes more active, and the development of the market attracts more financial institutions to enter the market, intensifying the competition among banks, which in turn leads to the reduction of lending interest rates or the increase of deposit interest rates in order to compete for market share, thus compressing the profit space of banks. The regression coefficients of the urbanization level and capital adequacy ratio are positive, and both regression coefficients pass the significance test at a 1% significance level. On the one hand, with the advancement of urbanization, infrastructure construction and residents’ consumption demand continue to grow, which in turn leads to an increase in the demand for financial services, and these demands provide more business opportunities and sources of income for commercial banks. On the other hand, the capital adequacy ratio is an essential indicator of a bank’s ability to withstand risks. A rise in the capital adequacy ratio means that banks have more capital to cover potential risk losses, thus improving their risk management capabilities. This helps to reduce the risk exposure faced by banks and safeguard their sound operations. Therefore, both urbanization level and capital adequacy ratio show a significant positive relationship with bank performance.

### 4.2 Mechanism analysis

Based on the study of hypothesis 2, the advancement of digital finance may increase the risk tolerance of financial institutions, such as banks, which, therefore, affects the performance of banks. Thus, this study aims to thoroughly examine the relationship between digital inclusive finance and bank performance by considering risk-taking as a mediating variable. To achieve this, a mediation effect model is constructed, and a specific regression analysis is conducted to assess the impact of digital inclusive finance on risk-taking, as outlined in Eq (4). The findings are displayed in Table 4 Model (1). Formulate Eq (5) to examine the impact of digital financial inclusion and risk-taking on bank performance. The findings are presented in Table 4, model (2).

**Table 4:** Analysis of regression results of the mechanism test.

Variables	Model (1)	Model (2)
	Risk-taking	ROE
**Risk-taking**		4.375***
		(6.67)
**Digital Financial Inclusion Index**	0.455***	3.857***
	(4.82)	(3.67)
**Economic development level**	-0.474**	-4.047*
	(-2.48)	(-1.97)
**Degree of industrial agglomeration**	-10.819**	45.123
	(-2.11)	(0.82)
**Urbanization level**	-0.454	38.248***
	(-0.46)	(3.63)
**Bank net interest margin**	-0.037	-1.198***
	(-1.42)	(-4.26)
**Capital adequacy ratio**	-0.017**	0.417***
	(-2.02)	(4.70)
**Constant**	-2.631**	0.393**
	(-2.50)	(2.02)
**Observations**	300	300
**R-squared**	0.407	0.788
**Number of id**	30	30

Note: (1) T-statistics are in parentheses. (2) ***, **, and * indicate significant at the 1%, 5%, and 10% significance levels, respectively.

The analysis of the findings from model (1) in the table indicates that the regression coefficient for digital finance development in relation to risk-taking is 0.455, which is statistically significant at the 1% level. This suggests that an increase in the level of digital finance development can enhance the bank’s risk-taking behaviour. With each unit of increase in digital finance development, the risk-taking ability will increase by 0.455%. This is primarily because the development of digital finance will expand the utilization of financial products and enhance the bank’s revenue sources, thereby facilitating the expansion of the bank’s size and the flow of surplus funds. Simultaneously, the advancement of digital inclusive finance is accompanied by a rise in the level of innovation and utilization of digital technology. This not only enhances the efficiency of banks in their management and operations, thereby increasing the operational risk they face but also enables precise analysis of the creditworthiness of customers and the restriction of credit amounts based on their credit status. Consequently, this enhances the risk-bearing capacity of the credit business.

The regression analysis of the model (2) reveals that the coefficient for the bank’s risk-taking ability on bank performance level is 4.375, with a significant level of 1%. This indicates that an increase in the bank’s risk-taking level leads to a noticeable improvement in the bank’s performance earnings. Furthermore, a higher risk-taking ability enables the bank to engage in medium and high-risk investments, increasing the likelihood of obtaining higher returns. In addition, improving the bank’s ability to handle risks can also serve as a constant driving force and assurance for advancing technological innovation and expanding the reach of inclusive finance, thereby elevating the bank’s performance. When considering the idea of the mediation effect, it is evident that the direct influence of digital financial inclusion on bank performance may be measured by the regression coefficient of 3.857 in the model (2).

Contrarily, based on the study of the coefficient of the model (1), it is evident that the indirect influence of the Digital Inclusion Index on the bank’s performance is the result of multiplying 0.455 by 4.375. The magnitude of the indirect impact of digital financial inclusion on the bank’s performance is 1.991. By considering the findings in Table 3, it is evident that the overall impact of digital financial inclusion on bank performance is 5.848. To summarize, the combined coefficients of the direct and indirect effects of digital financial inclusion on bank performance can be demonstrated through Hypothesis 2. Additionally, there is evidence of risk-taking playing a mediating role in the impact of digital financial inclusion on bank performance.

### 4.3 Heterogeneity test

The interpretation of the regression data in Table 3 reveals a general upward trend in bank performance as digital financial inclusion infrastructure develops. Despite this, China’s regions are marked by significant differences in scientific and technical advancement, resource endowment, and socioeconomic status. From a regional perspective, will enhancing digital financial inclusion for bank performance show different regional characteristics? Thus, this paper will utilize the standard of China’s geographical division to examine the province divided into the Eastern region, central region, western region, southern region, and Northern region, the digital financial inclusion, and the bank performance in each region separately. The results of the regression are displayed in Table 5.

**Table 5:** Analysis of regression results of heterogeneity test.

Variables	(1)	(2)	(3)	(4)	(5)
	Eastern Region	Central Region	Western Region	Northern Region	Southern Region
**Digital Financial Inclusion Index**	8.647***	10.996***	12.260***	11.098***	10.474***
	(13.37)	(13.80)	(15.23)	(18.51)	(15.73)
**Constant**	-60.697***	-72.661***	-79.875***	-72.283***	-71.834***
	(-16.86)	(-16.80)	(-18.41)	(-22.21)	(-19.62)
**Control variables**	Control	Control	Control	Control	Control
**Time**	Control	Control	Control	Control	Control
**Individual**	Control	Control	Control	Control	Control
**Observations**	110	80	110	150	150
**R-squared**	0.646	0.729	0.703	0.719	0.649
**Number of id**	11	8	11	15	15

Note: (1) T-statistics are in parentheses. (2) ***, **, and * indicate significant at the 1%, 5%, and 10% significance levels, respectively.

Upon examining the data in the table, it is evident that the regression coefficients of the digital finance index on bank performance are consistently positive and statistically significant at the 1% significance level. This suggests that the level of digital finance development has a noticeable positive impact on bank performance in different regions of China, enhancing it significantly. The Western region stands out among all regions in terms of the impact of digital finance on bank performance, as indicated by its regression coefficient of 1.455. This coefficient is statistically significant at the 1% level, suggesting that the level of digital finance development in the region has a highly noticeable effect on enhancing bank performance. The phenomenon can be attributed to the central features of digital financial inclusion, which are inclusiveness and digitization.

In contrast to the traditional financial model in rural and remote areas, which excludes the masses from accessing financial services, the digital financial inclusion model receives policy support from the government. This support mandates financial institutions to utilize digital technology to expand the reach of financial inclusion. The primary objective is to ensure the availability of digital finance in rural and other remote areas. The primary objective is to establish digital financial services in remote regions, mainly rural areas. China, being a major agricultural nation, has a significant proportion of its population (40.42%) engaged in farming. The western region, in particular, needs to catch up economically and has a substantial number of farmers and agricultural workers. The widespread adoption of digital inclusive finance in this region will significantly influence the performance of banks due to the advantages of a large population base, a significant number of individuals affected, and the backing of relevant national policies. Although the eastern region has a higher degree of economic and technological development, banks in this region may incur higher costs in order to enhance their research and development (R&D) capabilities and promote innovation in their operations and management. As a result, the impact on bank performance improvement is less pronounced compared to the western region. Based on the study provided, it is evident that there are notable variations in the influence of digital financial inclusion on bank performance across different regions. This finding supports Hypothesis 3.

### 4.4 Robustness check

The robustness test refers to verifying the credibility of the research results by regressing the variables by changing the measurement of the variables or replacing the model and analysing whether the regression results are consistent with the previous results. This paper conducts the robustness test by changing the bank performance measurement and replacing the double fixed effects model.

Table 6 displays the regression outcomes following the substitution of ROE with ROA and the replacement of the double fixed-effects model with the GMM model. The regression results demonstrate that the regression coefficients for the digital finance development level on bank performance, under both robustness tests, are 0.167 and 8.727, respectively. Both coefficients are statistically significant at the 1% level, indicating an apparent positive effect of improving the digital finance development level on bank performance. These results align with the benchmark regression results and provide evidence of the robustness of the findings.

**Table 6:** Analysis of robustness test regression results.

Variables	(1)	(2)
	Replace the dependent variable	GMM Model
**L.ROE**		0.332***
		(6.38)
**Digital Financial Inclusion Index**	0.167*	8.727***
	(1.91)	(3.60)
**Constant**	1.145*	138.40***
	(1.70)	(10.99)
**Control variables**	Control	Control
**Time**	Control	Control
**Individual**	Control	Control
**Observations**	300	270
**R-squared**	0.595	0.170
**Number of id**	30	30
**F-Statistics**	24.80***	9.01**
**Hansen-J**		23.81

Note: (1) T-statistics are in parentheses. (2) ***, **, and * indicate significant at the 1%, 5%, and 10% significance levels, respectively.

The regression results in Table 7 demonstrate the impact of each control variable on the relationship between the digital financial inclusion index and bank performance. Upon analyzing the data in the table, it is evident that the regression coefficient of the digital financial inclusion index on bank performance is 5.848. This coefficient is statistically significant at the 1% level, indicating that digital financial inclusion continues to have a significant positive effect on bank performance. The regression analysis reveals that the coefficient of economic development level on bank performance is -6.120, indicating a significant promotion effect at the 1% level. Additionally, the other control variables also exhibit varying degrees of influence on bank performance. This demonstrates that the outcomes of the durability assessment align with the outcomes of the standard regression, hence confirming the reliability of the acquired conclusions.

**Table 7:** Analysis of robustness test regression results.

Variables	(1)	(2)	(3)	(4)	(5)	(6)
**Digital Financial Inclusion Index**	10.797***	10.816***	10.836***	5.757***	5.304***	5.848***
	(24.17)	(24.34)	(24.37)	(5.03)	(4.82)	(5.38)
**Economic development level**		-4.654**	-4.232*	-6.824***	-6.315***	-6.120***
		(-1.99)	(-1.79)	(-2.92)	(-2.82)	(-2.79)
**Degree of industrial agglomeration**			-69.635	16.800	1.946	-2.210
			(-1.12)	(0.27)	(0.03)	(-0.04)
**Urbanization level**				55.712***	41.986***	36.264***
				(4.78)	(3.65)	(3.19)
**Bank net interest margin**					-1.513***	-1.361***
					(-4.95)	(-4.51)
**Capital adequacy ratio**						0.344***
						(3.62)
**Constant**	-72.127***	-28.838***	-31.053***	-15.028***	-4.905***	-11.116*
	(-29.55)	(-21.85)	(-21.94)	(-21.35)	(-20.57)	(-1.85)
**Observations**	300	300	300	300	300	300
**R-squared**	0.685	0.689	0.691	0.715	0.739	0.752
**Number of id**	30	30	30	30	30	30

Note: (1) T-statistics are in parentheses. (2) ***, **, and * indicate significant at the 1%, 5%, and 10% significance levels, respectively.

## 5. Conclusions and recommendations

### 5.1 Research findings

The integration of digital technology and financial inclusion has significantly accelerated the progress of digital financial inclusion, consequently augmenting the efficacy of financial services in rural and other geographically isolated regions. These entities’ combined efforts have successfully utilized digital financial inclusion capabilities to aid agricultural communities. Given the critical nature of the contemporary financial system, banks should take the lead in promoting and facilitating the dissemination of digital financial inclusion services, which would significantly contribute to expanding small and medium-sized enterprises (SMEs) and agriculture throughout the nation. Achieving greater digital financial inclusion will immediately and substantially affect the bank’s performance. This study draws upon pertinent data from 30 provinces in China from 2012 to 2021. Following the selection of variables, an analysis of the spatial distribution and development patterns of digital financial inclusion and bank performance in China during that period is conducted employing the kernel density estimation method. In conclusion, conclusions are drawn regarding the impact and mechanism by which digital financial inclusion influences bank performance. To summarize, the subsequent conclusions are deduced:

1. Digital financial inclusion and bank performance in China exhibit an upward trend annually. Nonetheless, variations in development rates can be observed across distinct regions. While these disparities become more pronounced beyond 2018, they do not exhibit polarization.
2. Enhancing digital financial inclusion can significantly enhance the degree of bank performance. Control variables such as economic development level, industrial agglomeration, and bank net interest margin can hinder the development of bank performance. Conversely, the level of bank performance can be considerably enhanced by urbanization level and bank capital adequacy ratio.
3. The efficiency of financial institutions is affected by the willingness to take risks, which mitigates the impact of digital financial inclusion. Improving the efficiency of financial institutions can be accomplished by promoting the widespread use of digital financial inclusion programs.
4. The impact of digital financial inclusion on bank performance varies significantly across different areas. The Northwest region stands out regarding the favorable influence of digital financial inclusion on bank performance. This is primarily due to the region’s strong emphasis on national policies and its substantial population of farmers. The heightened risks linked to lending operations in the central area negatively impact the bank’s performance. The impact of digital financial inclusion on bank performance is most pronounced in the eastern, northern, and southern regions.

### 5.2 Research recommendations

1. Maintain a high standard for building digital inclusive financial infrastructure, an essential prerequisite for any digital infrastructure. The use of digital technology to integrate financial resources, the establishment of industrial digital transformation funds, and the promotion of the transformation of small and medium-sized enterprises into digital industries are all ways to better construct digital financial inclusion facilities from the perspectives of businesses and individuals. Meanwhile, a high-coverage communication network should be established in rural areas so that all people may benefit from financial services, and regional disparities in the growth of digital financial inclusion can be gradually reduced.
2. To improve bank efficiency, it is necessary to enhance innovation in digital technology, broaden the range of financial products, and develop connectivity to large communication networks in underserved locations, such as rural areas. The credit system in our culture necessitates enhancement. Credit is a crucial cornerstone of the modern financial system, and its evolution throughout time has significant implications for banks and other financial institutions. It helps ensure the security of credit transactions and ensures the best operational efficiency of banks.
3. In order to increase customer acceptance and the efficiency of operations, banks must improve the innovation capacity of financial technology by concentrating on the regular updating of financial products and services and paying attention to the increasing degree to which digital technology is incorporated into the institution’s running. If necessary, banks can also provide staff with appropriate training in digital technology to streamline business processes and win over more clients.
4. The government needs to pay attention to the variations in regional resources and technical development that affect the efficiency of digital financial inclusion and banking. A focus on areas in China’s central region where digital financial inclusion has a negative impact on bank performance, followed by an investigation into the root causes and the implementation of appropriate policy deviations to spur the development of digital inclusive financial infrastructure there, aid in the enhancement of the region’s credit monitoring system, and bring it into conformity with the rest of the country are all necessary steps. Additionally, real-time policy adjustments and regional parity in development necessitate dynamic monitoring of the effects of digital financial inclusion and bank performance across the country.

### 5.3 Research limitations and future perspectives

This work utilizes the kernel density estimation methodology to analyse the spatiotemporal evolution characteristics and patterns of digital financial inclusion and bank performance in 30 Chinese provinces from 2012 to 2021. Subsequently, it utilizes the conventional panel model and mediation effect model to analyse the sequential pathway via which digital financial inclusion influences bank performance and the many pathways through which digital financial inclusion exerts its effects. Geographical differences exist in how digital financial inclusion affects bank performance. This study employs rigorous data analysis and empirical research to examine the correlation between digital financial inclusion and the development of bank performance. However, it is essential to note that some practical limitations need to be considered. Increasing the number of control variables in the regression model is impossible. Several elements have varying degrees of influence on the bank’s operational performance in enhancing the growth of bank performance. However, this paper primarily relies on the relevant literature to select control variables, considering a subjective perspective.

Therefore, in future research, we should determine the control variables in this paper through field surveys and interviews with experts. On the other hand, this paper focuses on risk-taking as a moderating element in investigating the influence of digital inclusive finance on bank performance. According to the literature, the mediating effect of digital inclusive finance on bank performance is influenced by various elements, including digital transformation, upgrading, economic agglomeration, industrial structure upgrading, and others. As a result, we will continue to investigate the path of the influence of digital inclusive finance on bank performance in depth in future studies to supplement the limits of the research in this work.

## Supporting information

S1 Data (XLSX)
